# AI‐Driven Defecation Analysis by Smart Healthcare Toilet: Exploring Biometric Patterns and Eu‐Tenesmus

**DOI:** 10.1002/advs.202503247

**Published:** 2025-05-11

**Authors:** Zhiquan Song, TaeHyung Kwon, Jeung Lee, Daeyoun D. Won, Brian J. Lee, Hyuk Soon Choi, Joseph C. Liao, Walter G. Park, Irene Sonu, Stephan Rogalla, Michael J. Rosen, David L. Hu, Jonathan Kuang Ziyang, Sunny Hei Wong, Bong Hyun Jun, Soh Kim, Seung‐min Park

**Affiliations:** ^1^ School of Chemistry Chemical Engineering and Biotechnology Nanyang Technological University Singapore Singapore; ^2^ Department of Civil and Environmental Engineering Stanford University Stanford CA USA; ^3^ Seokjeong Wellpark Hospital Jeollabuk‐do Republic of Korea; ^4^ Kanaria Health Seoul Republic of Korea; ^5^ School of Mechanical Engineering Sungkyunkwan University Suwon Republic of Korea; ^6^ Division of Gastroenterology and Hepatology Department of Internal Medicine Korea University College of Medicine Seoul Republic of Korea; ^7^ Department of Urology Stanford University School of Medicine Stanford CA USA; ^8^ Division of Gastroenterology & Hepatology Department of Medicine Stanford University School of Medicine Stanford CA USA; ^9^ Division of Pediatric Gastroenterology Hepatology and Nutrition Department of Pediatrics Stanford University School of Medicine Stanford CA USA; ^10^ George W. Woodruff School of Mechanical Engineering Georgia Institute of Technology Atlanta GA USA; ^11^ Department of Gastroenterology & Hepatology Tan Tock Seng Hospital Singapore; ^12^ Lee Kong Chian School of Medicine Nanyang Technological University Singapore Singapore; ^13^ Department of Bioscience and Biotechnology Konkuk University Seoul Republic of Korea

**Keywords:** biometrics, defecation, digital biomarker, smart toilet, tenesmus

## Abstract

Defecation, a fundamental physiological process, remains underexplored despite its importance in human health. To address this gap, a smart toilet system is developed that enables real‐time monitoring of defecation behaviors. Analyzing 45 defecation events from 11 participants, key defecation parameters are identified, including stool dropping duration, stool thickness, and eu‐tenesmus interval. Stool dropping duration follows a log‐normal distribution, with longer durations (>5 s) linked to lower Bristol Stool Form Scale (BSFS) scores, suggesting constipation (*p* = 0.008 for BSFS1/2/3 vs BSFS5/6/7). Stool thickness decreases with increasing BSFS scores (*p* = 5 × 10⁻⁶ for BSFS1/2/3 vs BSFS5/6/7), validating its role as an objective marker for bowel function. Eu‐tenesmus is introduced, defined as the interval between the last stool drop and cleansing, averaging 74.8 s. It shows significant gender differences (*p* = 0.014) but no correlation with stool consistency, suggesting its potential as an independent biomarker for gut health. Defecation behaviors between humans and animals is also compared in detail. Longitudinal monitoring demonstrates the potential for personalized health tracking and dietary recommendations. Furthermore, the feasibility of biometric identification is established using 11 defecation‐related parameters, including stool properties and cleansing behavior. These features enable high participant differentiation, supporting non‐invasive identity verification.

## Graphical abstract

A healthcare toilet system is introduced to passively measure defecation behavior. Real‐time data on stool dropping duration, thickness, and a newly defined “eu‐tenesmus” interval show correlations with stool form and gender differences. Results from 45 defecation events reveal a promising method for comprehensive defecation analysis. Defecation‐time and behavior metrics offer potential for biometric identification, illuminating personalized gut health monitoring.

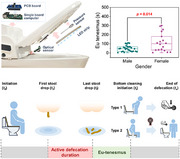

## 1 Introduction

The process of defecation, though an everyday natural phenomenon, plays a pivotal role in maintaining human health and well‐being. It is a key part of the body's waste disposal system, eliminating toxins and indigestible materials. Yet, despite the quotidian nature of this subject, comprehensive research investigating the patterns and intricacies of human defecation habits is extremely sparse.^[^ ^1^ ^]^ This lack of scientific investigation can be attributed in part to societal taboos,^[^ ^2^ ^]^ which have restricted open conversation and investigation into this essential physiological process. However, understanding the patterns and variability of human defecation habits could hold the key to a multitude of advancements in medical diagnostics,^[^ ^3^ ^]^ personalized health strategies, and dietary feedback.^[^ ^4^ ^]^ Furthermore, analyzing defecation behaviors can provide insight into broader aspects of human behavior, such as how individuals perceive and manage bodily functions, interact with their environments, and adhere to social norms regarding privacy and hygiene. These insights are crucial for developing interventions that improve health outcomes and address social challenges related to public health and sanitation. The potential implications of this understanding could lead to significant improvements in precision health^[^ ^5^ ^]^ and overall quality of life (e.g., constipation management).^[^ ^6^ ^]^

The recent advancements in technology, specifically in the field of health monitoring, have opened new opportunities to gather and analyze data on human defecation behaviors. Smart toilet systems, with their ability to monitor and evaluate human excretory patterns in real time, provide an unprecedented platform for data collection.^[^ ^7^ ^]^ These systems are non‐invasive and convenient, offering an ideal solution for conducting large‐scale longitudinal studies and data analysis. As proposed in our prior perspective.^[^ ^5c^ ^]^ human excreta, specifically urine and stool, hold a myriad of “analogue” biomarkers that could potentially transition to digital biomarkers^[^ ^1^ ^]^ for more precise monitoring. A prominent example is the Bristol Stool Form Scale (BSFS),^[^ ^8^ ^]^ a visual classification of stool morphology employed in the Rome diagnostic criteria^[^ ^9^ ^]^ for Irritable Bowel Syndrome (IBS).^[^ ^10^ ^]^ Nonetheless, the current “analogue” diaries^[^ ^11^ ^]^ are challenged by variations in BSFS interpretation^[^ ^12^ ^]^ by patients and a general lack of enthusiasm in consistent stool recording. Moreover, patients may struggle with recall bias, often unable to accurately remember and document their stool characteristics. The introduction of objective and automated methods, leveraging computer vision and artificial intelligence within smart toilet systems, could transition these analogue biomarkers into digital ones. These could offer more comprehensive and reliable data for clinical interpretation, circumventing the inaccuracies tied to patient‐reported outcomes.

**Figure 1:**
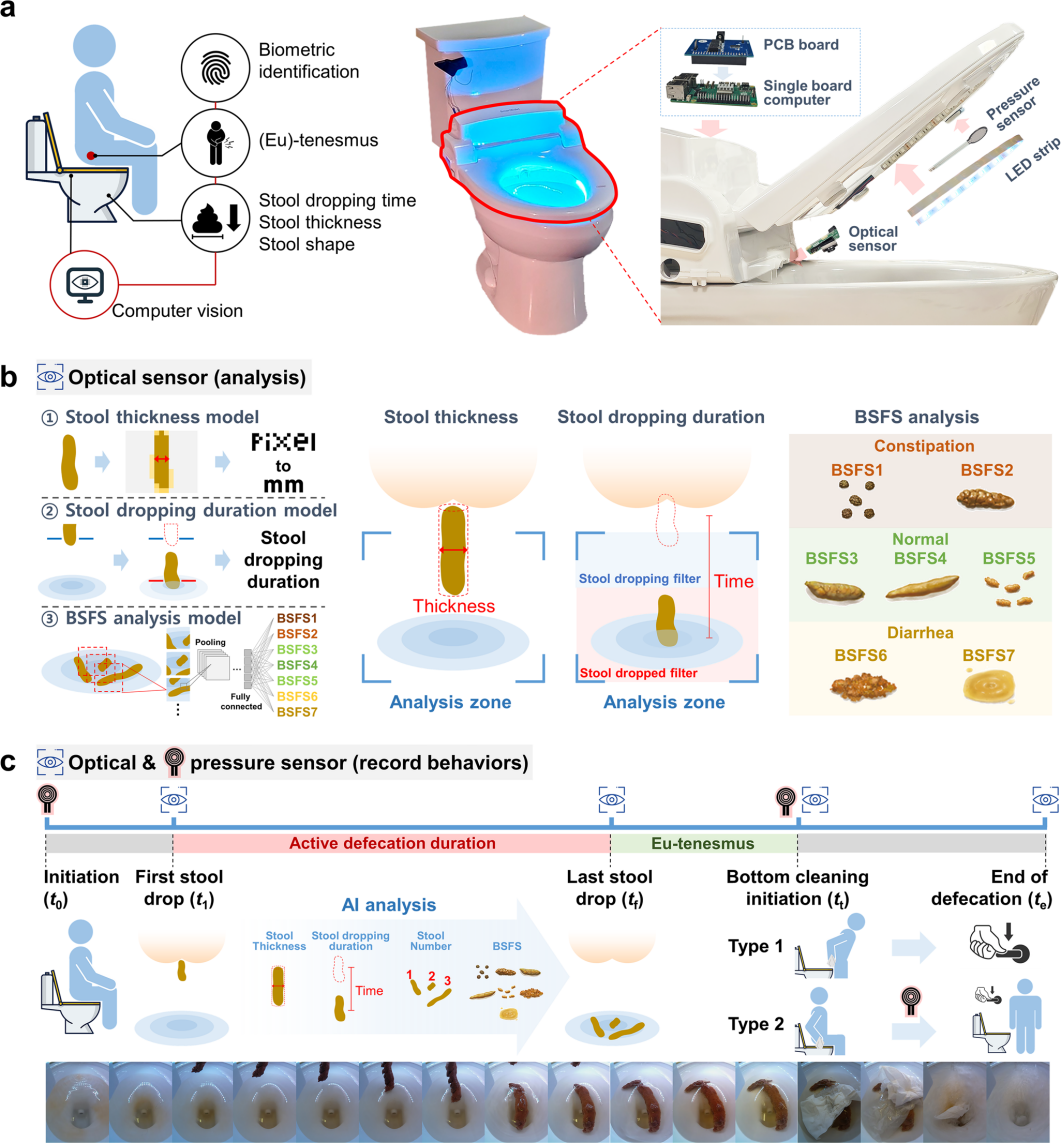
Schematic illustration of the smart toilet's working mechanism. a) The mountable smart toilet system comprises an optical sensor, a pressure sensor, a light‐emitting diode (LED) strip, and a single‐board computer, all connected via a printed circuit board (PCB). This configuration enables real‐time monitoring of defecation conditions and behaviors. b) The optical sensor supports three analytical models for defecation assessment. 1) Stool thickness model: the stool is detected in the captured image, and its thickness is calculated by converting pixel counts to millimeters, applying a transformation coefficient based on the distance between the stool and the sensor. 2) Stool dropping duration model: Two filters, “stool dropping” and “stool dropped,” track the stool while it is being passed and after it has fallen into the toilet, respectively. By computing the time difference between these detections, it is possible to determine the stool dropping duration and to pinpoint both the first and last stool events. 3) Bristol stool form scale (BSFS) model: A convolutional neural network (CNN) classifies the stool into seven categories. BSFS1 and BSFS2 for constipation, BSFS3 to BSFS5 for normal stool, and BSFS6 and BSFS7 for diarrhea. c) By integrating temporal data from the optical sensor and pressure sensor, the system can further derive key parameters such as the first and last stool drop times, active defecation duration, eu‐tenesmus, and other defecation‐related behaviors.

In this work, we employ the capabilities of a smart toilet system to analyze the defecation behaviors, as shown in **Figure** **1**a. A total of 45 defecation events were examined to provide an in‐depth understanding of the dynamics and variability involved in defecation. More details are provided in Table S1 (Supporting Information). Our focus was not only on the nature and consistency of the stool but also on the timing, frequency, and duration of defecation events—all of which can pave the way to an individual's health monitoring and digestive efficiency. An intriguing finding from our investigation is the emergence of a subcategory of tenesmus sensation, termed “eu‐tenesmus.” Derived from the Greek “eu,” meaning “good” or “healthy,” it describes the period between the last stool dropping and the cleansing behavior. This variant, identified through the precise measurement of the time between the last stool dropping and toilet paper usage, presents a novel aspect of normal bowel physiology and introduces new potential for health monitoring.^[^ ^13^ ^]^

The parameters that can be obtained from smart toilet system, such as stool thickness and BSFS, not only provide standardized metrics for assessing stool health but also facilitate real‐time monitoring to minimize recall bias. These measurements, coupled with our novel concept of “eu‐tenesmus”, enhance our understanding of gastrointestinal function by transforming subjective sensations into quantifiable digital biomarkers. Importantly, previous studies have shown that women are at a higher risk of developing functional bowel disorders, including constipation and pelvic floor dysfunction, which can manifest as extended defecation times and altered stool thickness. Notably, a meta‐analysis found that the ratio of women to men in these conditions is approximately 1.4 to 1.6:1.^[^ ^14^ ^]^ Furthermore, women exhibit more frequent reports of straining and incomplete evacuation,^[^ ^15^ ^]^ higher rates of obstructed defecation (e.g., 33.2% in one study^[^ ^15^ ^]^), and are more likely to show abnormal balloon expulsion times, indicative of pelvic floor dysfunction.^[^ ^16^ ^]^ Population‐based surveys have also revealed that a greater proportion of women defecate less frequently than daily, suggesting a shift toward the constipated end of the spectrum.^[^ ^17^ ^]^ Hence, continuous tracking of these stool parameters—thickness, BSFS type, and the eu‐tenesmus interval—allows for the investigation of potential gender‐specific variations and aids in establishing benchmarks for gastrointestinal conditions such as IBS and pelvic floor disorders.

In terms of longitudinal monitoring, the smart toilet system was leveraged to conduct continuous surveillance of defecation events. This approach allowed us to track changes, and detect potential anomalies alongside health issues over time, thereby painting a more holistic and accurate picture of human excretory behavior. Furthermore, we investigated the variability in stool excretion patterns across different individuals, finding that this diversity could offer valuable insights for the development of personalized medical interventions and dietary recommendations. Our study also ventured into a less‐explored territory of using defecation habits for biometric identification. We found that certain unique traits associated with defecation, such as the duration of the process, the techniques of bottom‐wiping, the timing of the first stool drop, and other parameters could be used to accurately identify individuals. This novel application of biometrics has wide‐ranging implications, particularly for privacy and personalized health monitoring. These findings not only confirm the existence of a largely untapped source of health‐related data but also highlight the ability of smart toilet systems to convert this data into valuable digital biomarkers. We believe that these findings could revolutionize diagnostics and treatment strategies, leading to more personalized and effective health management strategies.

The use of smart toilet systems goes beyond mere data collection and provides a pathway toward actionable insights for health monitoring, diagnostics, and personalized medicine. By integrating stool consistency, defecation timing, and related indicators with broader health metrics, clinicians can create comprehensive patient profiles that capture subtle changes in gastrointestinal or metabolic status. As highlighted by Wang et al.,^[^ ^13^ ^]^ combining these data streams with other relevant biomarkers enables a precision health approach, leveraging real‐time monitoring to offer immediate feedback and timely interventions. Furthermore, the application of force‐sensitive pressure sensors, computer vision, and artificial intelligence techniques allows for continuous surveillance of bowel habits, early detection of functional bowel disorders, and nuanced assessments of pelvic floor dysfunction. These insights pave the way for personalized dietary and therapeutic recommendations, complementing existing microbiome research to yield holistic, longitudinal views of patient health. As such, the development of predictive models—validated in collaboration with gastroenterologists—can help stratify risks for conditions like IBS or colorectal cancer, ultimately guiding clinical decision‐making. Addressing data privacy concerns, improving user comfort, and ensuring seamless system integration will be pivotal in advancing user acceptance and scaling this technology. Overall, these advancements mark a paradigm shift in how routine bodily functions can be transformed into meaningful, personalized data for improved health outcomes.

## 2 Experimental Section

### 2.1 Overview of the Smart Healthcare Toilet System

The “smart toilet” prototype, recently featured in a publication,^[^ ^7^ ^]^ was developed and tested at the Clark Center at Stanford University for the purpose of analyzing human biospecimens such as urine and stool. The research device, installed in compliance with the institutional review board (IRB) regulations, was operational only during participant usage, as this study was approved by the Stanford IRB (Protocol#: 45 621). This system offers multiple functions; however, this study emphasizes its capability to continuously photograph stool samples and classify them on the BSFS with the help of an automated machine learning‐based classifier developed by our team. Additionally, the prototype was designed to record the time to first stool and total seated time, metrics that were crucial for medical professionals managing conditions like constipation, pelvic floor dysfunction, and IBS, using computer vision and pressure sensors. While the system included user identification features, such as fingerprinting and unique anodermal attributes, these were not utilized for participant identification in this research. Currently, 11 adult participants were recruited, all volunteers without any apparent gut‐related symptoms, as the only exclusion criterion for this study was the age limitation—no minors were included. The data collected from these participants was retrospectively analyzed and were elaborated upon in this article.

### 2.2 Defecation Monitoring

In this study, a commercially available electronic bidet (BioBidet, Bemis Manufacturing Co., Sheboygan Falls, WI, USA) was salvaged, where a mountable smart toilet system was made using the external casing. As shown in Figure 1a, the mountable smart toilet system comprised the following components: an optical sensor (Raspberry Pi Camera Module 3, Raspberry Pi Foundation, Cambridge, England, UK) located in the opening previously occupied by the bidet nozzle; a pressure sensor (FSR 402, Interlink Electronics, Irvine, CA, USA) positioned at the front of the toilet seat; an light‐emitting diode (LED) strip integrated into the inner ring of the seat; a single‐board computer (Raspberry Pi v3, Raspberry Pi Foundation, Cambridge, England, UK); and a customized printed circuit board (PCB) that connected the sensors and LED strip to the single‐board computer. This configuration enabled real‐time monitoring of defecation conditions and behaviors.

An optical sensor and artificial intelligence (AI) models were used to analyze stools, as illustrated in Figure 1b. There were three stool analysis models for defecation assessment:

#### 2.2.1 Stool Thickness Model

The stool was detected in the captured image, and its thickness was calculated by converting pixel counts to millimeters, applying a transformation coefficient based on the distance between the stool and the sensor. In practical scenarios, as the distance between an object and the camera increased, the object's apparent size in the image decreased. To accurately correlate spatial data, this study computed a transformation coefficient. Specifically, three representative stool‐dropping locations—rear, center, and front—were identified relative to the participant's seating position, as shown in Figure S1 (Supporting Information). These patterns were determined based on the most frequently observed average positions, which reflected distinct participant behaviors. The primary objective was to calculate the stool thickness at the point of contact with the water. To precisely assess spatial dimensions within the current imaging setup, the following measurements and calculations were performed.

Utilizing the Raspberry Pi's specified horizontal field of view (FOV), the real‐world horizontal dimension captured in the image at a known distance from the camera can be calculated as follows.

(1) $$D_{hor}=2d\;\tan\left(\frac{\mathrm{Fo}V_{hor}}{2}\right)$$

where *D*_hor_ is the real‐world horizontal dimension, *d* is the distance from the camera, and FOV_hor_is the angle of the specified horizontal field of view. Based on the ratio of the stool's horizontal pixel width to the total image width, the stool thickness can be calculated as follows.

(2) $$D_{stool}=2d\;\tan\left(\frac{\mathrm{Fo}V_{hor}}{2}\right).\left(\frac{P_{stool}}{Pimg}\right)$$

where *D*_stool_ is the stool thickness *P*_stool_ is the horizontal pixel of the stool, and *P*_img_ is the total horizontal pixel of the image. The distances between the camera and the stool‐dropping locations (*d*) were measured for the rear, center, and front positions, which were 193, 237, and 290, respectively. These three different locations in the camera view have different vertical pixel counts. Based on the vertical pixel count, a transformation coefficient was calculated as follows.

(3) $$y_{\mathrm{coeff}}=0.092840\times \left(0.000037x^{1.497}+1\right)$$

where *x* is the vertical pixel count from the bottom of the image to the stool‐dropping location, and *y*_coeff_is the transformation coefficient. By multiplying *y*_coeff_ with the stool thickness in pixels, the stool thickness in millimeters can be calculated.

#### 2.2.2 Stool Dropping Duration Model

Two filters, “stool dropping” and “stool dropped,” track the stool while it was passed and after it was fallen into the toilet, respectively. By computing the time difference between these detections, it is possible to determine the stool‐dropping duration and to pinpoint both the first and last stool events. Each image was named using the format *hhmmss.ssss* (e.g., 135958.5042.jpeg corresponds to 13:59:58.5042 in 24 h clock format), enabling precise calculation of capture times and intervals.

#### 2.2.3 BSFS Analysis Model

A deep convolutional neural network (CNN) trained on over 2500 clinician‐scored stool images provided robust stool classification based on the BSFS. This classifier achieved an area under the curve (AUC) exceeding 0.90, ensuring reliable differentiation among constipation (BSFS 1–2), normal (BSFS 3–5), and diarrhea (BSFS 6–7) stool forms.

By integrating temporal data from the optical and pressure sensors, the system enabled comprehensive monitoring of the entire defecation process. The timeline of this process is illustrated in Figure 1c. When a participant sat on the seat of the smart toilet system, the pressure sensor detected their presence, and the LED strip was activated, providing sufficient illumination for the optical sensor to analyze the stool. The time at which the participant sat down was recorded as *t*_0_. A custom‐trained *toilet state model* classified the toilet's state as clean, urine, stool, or toilet paper, enabling further analysis of the sequence of events, such as whether urination occurred before or after defecation. The first stool‐dropping time was determined when the stool‐dropping filter of *stool dropping duration model* identified stool and this was double confirmed by the *toilet state model*, which transitioned from a clean or urine state to a stool state. This time was recorded as *t*_1_.

During the active defecation phase, three analytical models (*stool thickness model*, *stool dropping duration model*, *and BSFS analysis model*) were employed to evaluate stool properties. These models provided key parameters such as stool thickness, stool dropping time, stool count, and BSFS grade. The last stool‐dropping duration was detected by the *stool dropping duration model* when the final stool was identified, which was then labeled as *t*_f_. Throughout this active defecation period, data acquisition was conducted using the optical camera.

Following defecation, the bottom cleaning behavior was recorded using both the optical and pressure sensors. The cleaning start time was determined by the *toilet state model* transitioning from stool state to toilet paper state, and this time was labeled as *t*_t_. The cleansing type was defined based on pressure sensor data. If the pressure sensor returned to its initial state before detecting toilet paper, the participant cleaned while standing. Conversely, if toilet paper was detected before the pressure sensor returned to its initial state, the participant cleaned while seated.

Finally, the end of the defecation process was defined as the moment when the *toilet state model* indicated a transition to a clean state. This time was recorded as *t*_e_. Data for this phase were obtained through the optical sensor. Several parameters were defined to quantitatively evaluate the defecation process, including active defecation duration, total defecation duration, and eu‐tenesmus. The calculation formulas are as follows:

(4) $$\mathrm{Activate}\;\mathrm{defecation}\;\mathrm{duration}=t_{f}-t_{1}$$

(5) $$\mathrm{Total}\;\mathrm{defecation}\;\mathrm{duration}=t_{e}-t_{0}$$

(6) $$\mathrm{Eu}-\mathrm{tenesmus}=t_{t}-t_{f}$$

### 2.3 Study Participants

A group of 11 participants, comprising 5 females and 6 males, was recruited with ages ranging from 19 to 41 years, for a system testing phase spanning 5 weeks. There was only one exclusion criterion—no minors in this study. The participants were instructed to interact with the toilet system in the same manner as they would with a regular toilet, thereby maintaining natural defecation behavior, while they were recommended not to use the smart devices. Each participant contributed multiple data points through several defecation events, with each participating in up to 10 individual events. This resulted in a total of 55 recorded defecation events, providing a substantial dataset for analysis. For a comprehensive overview of these collected and measured values, please refer to Table S1 (Supporting Information).

### 2.4 Statistical Analysis

All statistical analyses were conducted to rigorously evaluate defecation‐related parameters (e.g., stool thickness, stool dropping duration, eu‐tenesmus). Each continuous variable was first assessed for normality (e.g., via the Shapiro–Wilk test), and an interquartile range (IQR)–based method (IQR multiplier = 1.5) was employed to remove outliers without assuming a normal distribution. For variables meeting normality criteria, independent *t*‐tests (e.g., Welch's *t*‐test) were performed to compare groups (e.g., gender differences), whereas non‐normally distributed data were analyzed using the Mann–Whitney U test. All comparisons were two‐sided with a significance level (*α*) of 0.05. Unless otherwise indicated, continuous variables were presented as mean ± standard deviation (SD), and categorical variables (e.g., cleansing type: seated vs standing) are reported as counts or percentages. A total of 55 defecation events from 11 participants were initially recorded, and after applying the IQR‐based outlier detection procedure, 45 valid samples remained for final analyses. Each statistical comparison explicitly noted the number of included samples, and the sample size (*n*) for each analysis reflected the observations after outlier exclusion. Statistical computations were performed in Python (version 3.9), primarily utilizing SciPy (version 1.9.1) and NumPy (version 1.23), with pandas (version 1.4) for data handling and matplotlib (version 3.5) for visualization.

## 3 Results

### 3.1 Log‐Normal Distribution of Stool Dropping Duration

**Figure 2:**
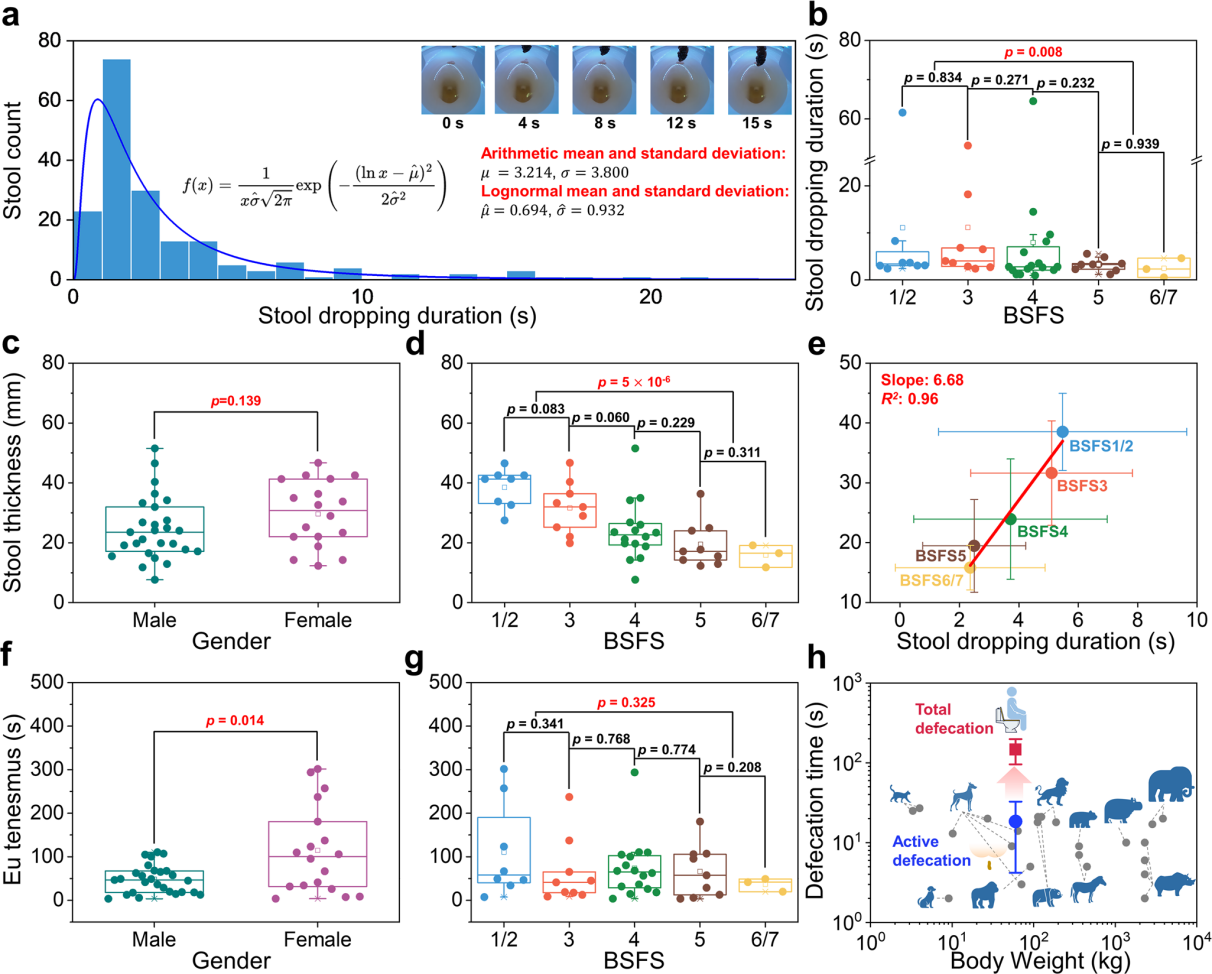
Comprehensive analysis of defecation dynamics and comparative zoological patterns (*n* = 45; statistical significance was assessed using either a two‐sided Welch's *t*‐test or Mann–Whitney U test depending on normality; differences with *p* < 0.05 were considered statistically significant). a) Distribution of stool dropping duration among study participants, highlighting significant variability and potential indicators of gastrointestinal health. b) Stool‐dropping duration comparison between BSFS scores. There is no significant difference in stool‐dropping duration between adjacent BSFS scores, but there is a statistically significant difference between constipation and diarrhea. c) Stool thickness comparison between male and female. There is no significant difference between males and females. d) Stool thickness comparison between BSFS scores. Stool thickness increases as the BSFS score gets lower. e) The relationship between stool dropping duration and the stool thickness. f) Eu‐tenesmus comparison between male and female. There is a significant difference between males and females. g) Eu‐tenesmus comparison between BSFS scores. There is no significant difference between BSFS scores. h) A comparative analysis of human and zoological defecation patterns shows that the total defecation duration in humans is significantly longer than in animals, but the active defecation duration is similar.

We utilized the advanced functionalities of the smart toilet to monitor stool‐dropping duration. This measurement can help identify individuals who may have pelvic outlet obstruction, straining, or other related conditions. Stool dropping duration is defined as the time required for stool to travel from the rectum (i.e., anal opening) to the waterline of the toilet bowl. To calculate this duration, the water surface level was determined to be approximately 1300 pixels on average. In our detection system, the region above 1300 pixels is processed by the stool‐dropping filter, while the region below 1300 pixels is processed by the stool‐dropped filter. By calculating the time difference between when the stool is first detected by these two filters, the stool‐dropping duration is obtained. As shown in **Figure** **2**a, the collected data is presented as a histogram, which has been fitted with a lognormal curve. The arithmetic mean (μ) and standard deviation (σ) of the dataset are 3.214 and 3.800, respectively. Our stool‐dropping duration data is best represented by the log‐normal distribution. This statistical model is skewed, peaking around the mean with a considerable tail – a pattern representing our collected data. For the lognormal distribution, the mean ($\hat{\mu }$) and standard deviation ($\hat{\sigma }$) are 0.694 and 0.932, respectively. The corresponding lognormal probability density function is given by:

(7) $$f\left(x\right)=\frac{1}{x\hat{\sigma }\sqrt{2\pi }}\exp\left(-\frac{{\left(\mathrm{In}\;x-\hat{\mu }\right)}^{2}}{2{\hat{\sigma }}^{2}}\right)$$

The average stool‐dropping duration is occurring around the 3.214 s, however, there is a noticeable tail extending up to 22 s. The per‐participant average stool‐dropping duration ranges between 1 to 5 s, indicating a significant variability in stool‐dropping duration with a generally short average duration of under 3 s. This pattern aligns with our observations, wherein the majority of stool‐dropping durations are relatively brief. However, a small but notable subset exhibits prolonged durations.

As illustrated in Figure 2b, all instances of stool dropping durations exceeding 5 s correspond to BSFS1–4, indicating that longer dropping durations are generally associated with lower BSFS scores. This is because stool with a lower BSFS score has low viscosity and a high friction coefficient, which causes it to adhere more easily to the anus. The *p*‐values for the comparisons between BSFS1/2 and BSFS3, BSFS3 and BSFS4, BSFS4 and BSFS5, as well as BSFS5 and BSFS6/7 were 0.834, 0.271, 0.232, and 0.939, respectively, indicating no statistically significant differences among these adjacent BSFS scores. However, the comparison between BSFS1/2/3 and BSFS5/6/7 yielded a *p*‐value of 0.008, demonstrating a statistically significant difference in stool‐dropping duration at the 95% confidence level (*α* = 0.05). This finding not only illustrates the inherent variability in defecation behavior but also provides a robust statistical framework for distinguishing between normal and pathological patterns.

### 3.2 Varying Stool Thickness of Individuals

In this study, leveraging a novel computer vision algorithm, the thickness of each stool specimen was quantified by computing the indices of the object's horizontal extremities as shown in Figure S1 (Supporting Information). We introduce a transformation coefficient to convert pixel measurements into millimeters. This coefficient varies depending on the distance between the stool and the optical sensor and the angle of optical sensor. It is determined by comparing the size of a known object at different positions within the image. The conversion equation is given by:

(8) $$T=0.092840\times \left(0.000037x^{1.497}+1\right)\times P$$

where *T* represents the stool thickness in millimeters, *x* denotes the pixel count from the bottom of the image to the stool's dropping location (which depends on the participant's sitting position), and *P* is the pixel count corresponding to the stool thickness. Results indicated an average thickness of 26.6 mm, with a substantial standard deviation of 10.9 mm. A thorough dataset analysis segmented by various parameters elucidated patterns in human defecation behaviors. As shown in Figure 2c, gender‐wise categorization showed males averaging 24.6 mm (Standard Deviation [SD]: 10.5 mm) and females 29.6 mm (SD: 11.0 mm). Utilizing Welch's *t*‐test, suitable for disparate sample sizes, revealed no significant gender difference in stool thickness at a 95% confidence level (*α* = 0.05), with a *p*‐value of 0.139. Although the difference in stool thickness between males and females was not statistically significant, the mean stool thickness for females was higher than that for males. This finding suggests that gender differences in stool thickness could still be considered when establishing criteria for evaluating normal stool thickness.

Subsequently, we investigated the relationship between stool thickness and the BSFS score. As shown in Figure 2d, the *p*‐values for comparisons between BSFS1/2 with BSFS3, BSFS3 with BSFS4, BSFS4 with BSFS5, and BSFS5 with BSFS6/7 are 0.083, 0.060, 0.229, and 0.311, respectively, indicating no statistically significant differences among these adjacent BSFS scores. However, the *p*‐value for the comparison between BSFS1/2/3 and BSFS5/6/7 is 5 × 10^−6^, indicating a statistically significant difference in stool thickness at the 95% confidence level (*α* = 0.05). The mean stool thickness for BSFS1/2, BSFS2, BSFS3, BSFS4, BSFS5, and BSFS6/7 are 38.5 mm (SD: 6.4 mm), 31.6 mm (SD: 8.7 mm), 23.9 mm (SD: 10.0 mm), 19.5 mm (SD: 7.7 mm), and 15.8 mm (SD: 3.7 mm), respectively. These results suggest that while stool thickness alone cannot precisely determine the BSFS score, it can aid in diagnosing constipation and diarrhea. Furthermore, our findings indicate that the BSFS score strongly influences stool thickness, as stool thickness decreases with increasing BSFS scores. Previous research has shown that women are more likely to report symptoms associated with constipation and pelvic floor dysfunction, including straining during defecation and feelings of incomplete evacuation.^[^ ^14^, ^15^ ^]^ Figure 2c illustrates that females tend to have thicker stool, and Figure 2d shows that stool with lower BSFS scores is also thicker. This finding aligns with prior research suggesting that women are more prone to constipation.

The relationship between stool‐dropping duration and stool thickness is shown in Figure 2e. Stool thickness exhibits a linear increase with stool dropping duration, with a slope of 6.68 mm s^−1^ and an *R^2^* value of 0.96. In Figure 2b, we observed that lower BSFS scores tend to be associated with longer stool‐dropping durations, while Figure 2d indicates that lower BSFS scores correspond to thicker stool. These three findings mutually validate each other, demonstrating that our sensors and AI models perform accurately and provide statistically meaningful data. Furthermore, our results align well with previous research, confirming that our smart toilet system is suitable for studying human defecation behavior.

### 3.3 Tenesmus in Healthy Individuals (Eu‐Tenesmus)

With a well‐trained AI model and a reliable hardware system, our smart toilet system aims to measure and study tenesmus. Tenesmus is the sensation of incomplete evacuation of the rectum, even after the completion of a bowel movement.^[^ ^18^ ^]^ It is a common symptom of various medical conditions, including constipation, IBS,^[^ ^19^ ^]^ inflammatory bowel diseases (IBD),^[^ ^20^ ^]^ and rectal cancer. However, data from our participants indicated, even in healthy populations, noticeable delays between the last stool dropping and the completion of their defecation activities (e.g., participants commencing post‐defecation clean‐up). Consequently, we have defined a new concept, “eu‐tenesmus” to describe the period between the last stool dropping and the cleansing behavior.^[^ ^21^ ^]^ Our findings revealed an average eu‐tenesmus duration of 74.8 s (SD: 74.9 s), ranging from 3.6 s to 301.7 s. As shown in Figure 2f, it was observed that females typically had a longer duration of tenesmus than their male counterparts, with mean durations of 114.6 s (SD: 100.6 s) for females and 48.2 s (SD: 32.4 s) for males. The *p*‐value for the eu‐tenesmus of male and female is 0.014, indicating a statistically significant difference at the 95% confidence level (*α* = 0.05).

Interestingly, as shown in Figure 2g, our results revealed no significant association between BSFS and eu‐tenesmus duration. The *p*‐values for comparisons between BSFS1/2 and BSFS3, BSFS3 and BSFS4, BSFS4 and BSFS5, BSFS5 and BSFS6/7, and BSFS1/2/3 and BSFS5/6/7 were 0.341, 0.768, 0.774, 0.208, and 0.325, respectively. These *p*‐values indicate that eu‐tenesmus duration does not significantly differ across different BSFS scores at the 95% confidence level (*α* = 0.05). These results suggest that eu‐tenesmus duration differs significantly by gender but does not correlate with BSFS scores. This finding implies that eu‐tenesmus duration may serve as an important digital biomarker for assessing gut health, independent of stool consistency (e.g., constipation or diarrhea). It appears to be more closely related to pelvic floor dysfunction, including straining during defecation and feelings of incomplete evacuation, conditions that have been reported to occur more frequently in women.^[^ ^14^, ^15^ ^]^ These observations indicate that eu‐tenesmus captures a distinct aspect of bowel function that may not be apparent from stool form alone. By translating the subjective sensation of completion (or lack thereof) into a quantifiable metric, eu‐tenesmus has the potential to reveal early indicators of functional bowel disorders, such as rectal hyposensitivity, outlet obstruction, or IBS, even among individuals who do not exhibit abnormal stool consistency. Furthermore, integrating eu‐tenesmus data with continuous smart toilet measurements and broader behavioral factors may help clinicians develop more personalized and proactive treatment strategies, particularly for mild or subclinical cases, where symptoms have yet to become overt.

We believe that our findings are important because they provide objective data on tenesmus in healthy individuals suggesting that this eu‐tenesmus could act as a digital biomarker for understanding pathological tenesmus. However, it is noteworthy that our study was limited by the small number of participants (*n* = 11). Future studies with a larger number of participants are necessary to confirm our findings and to better understand the factors that affect tenesmus and eu‐tenesmus in a population‐wide setting. This includes study participants of different ethnicities, ages, and genders for better representation. In addition, future studies should also investigate the impact of lifestyle factors on eu‐tenesmus, such as diet, exercise, stress, and the usage of digital devices in defecation. This information could be used to develop preventive strategies for tenesmus and to improve the quality of life for people who experience this symptom.

### 3.4 Difference in Defecation Patterns in Humans and Animals

Defecation patterns can be used to detect disease in both humans and animals. There is much data on domesticated cats because their owners regularly clean their litter boxes. Approximately 85% of healthy cats defecate one or more times per day, while approximately 15% defecate less than once per day. In contrast, among cats with chronic kidney disease, 58% defecate at least once daily, whereas 42% defecate less than once per day.^[^ ^22^ ^]^ Given this striking reduction in defecation frequency, there is the potential for automated detection of disease in cats using self‐cleaning litterboxes. There is much interest in cat defecation patterns because problems with waste elimination can influence adoption rates from shelters. Additionally, defecation patterns in cats may also be influenced by litterbox size.^[^ ^23^ ^]^

Some information is available about the other properties of feces across animals, such as density, number of pieces produced, and size. Yang et al.^[^ ^24^ ^]^ showed that density is a good indicator of diet, with carnivores producing sinkers and herbivores producing floaters. Previous studies, including that conducted by Yang et al.,^[^ ^24^ ^]^ have also examined the defecation characteristics of various animals, ranging from cats to elephants. Their findings suggest a nearly constant defecation duration of 12 ±  7 s across mammals with rectum lengths from 4 to 40 cm. The factors that influence defecation in animals are complex and may include factors such as social rank.^[^ ^25^ ^]^ Similarly, humans have similar complex influences on their defecation patterns: they have significant variability in stool form, frequency, and evacuation dynamics, influenced by multiple factors such as diet, stress, lifestyle, and underlying health conditions.

Our findings indicate a significant deviation from the defecation patterns observed in other heterotrophic organisms. Specifically, we observed that the average total defecation duration in humans, including cleaning time, is approximately 147.1 s (SD: 51.3 s). This duration is substantially longer than that reported in other mammals, as illustrated by the symbol ● in Figure 2h. Furthermore, the active defecation duration (defined as the duration between the first and last stool‐dropping time) was recorded at 18.4 s (SD: 14.3 s), represented by the symbol ▲. This duration closely aligns with, or is even shorter than, that observed in other mammals.

These findings suggest that the extended total defecation time in humans is not solely dictated by physiological constraints but may also be influenced by psychological and social factors.^[^ ^26^ ^]^ The variability in defecation patterns, coupled with the prolonged total duration, further implies that human bowel habits may be modifiable through interventions such as training (e.g., “potty‐training”) or biofeedback. This suggests that defecation behaviors are not exclusively governed by physiological processes but can also be shaped by adaptive behaviors.^[^ ^27^ ^]^ In humans, normal defecation frequency varies widely, with a range from three times per day to three times per week considered normal. This variability overlaps with that observed in mammals, yet the additional behavioral and environmental influences in humans differentiate human defecation patterns from those of other species.

Our smart toilet system offers a novel and reliable platform for long‐term, continuous monitoring of defecation patterns. By accurately recording and analyzing defecation duration, stool characteristics, and user behaviors—using a pressure sensor and an optical sensor that analyzes only the toilet bowl without capturing any part of the body, along with a trained AI model—this system not only provides insights into gastrointestinal health but also has the potential to detect and interpret broader physiological and psychological parameters, such as stress levels and habitual changes. Through prolonged data collection and advanced analytics, it becomes feasible to track behavioral shifts over time. Investigating factors such as lifestyle habits, educational background (e.g., toileting practices), and whether these behaviors evolve through training or environmental influences. Accordingly, the future applications of our smart toilet extend beyond gastrointestinal assessments, enabling a more comprehensive evaluation of user health. With ongoing research and refinement, this technology may serve as a valuable tool for personalized health monitoring, early disease detection, and the promotion of long‐term well‐being. Furthermore, we believe that it can also contribute to a deeper understanding of eu‐tenesmus, which is considered to be influenced not only by stool morphology but also by other factors, as we anticipated in Section 3.3.

### 3.5 Longitudinal Observations and Health Implications

**Figure 3:**
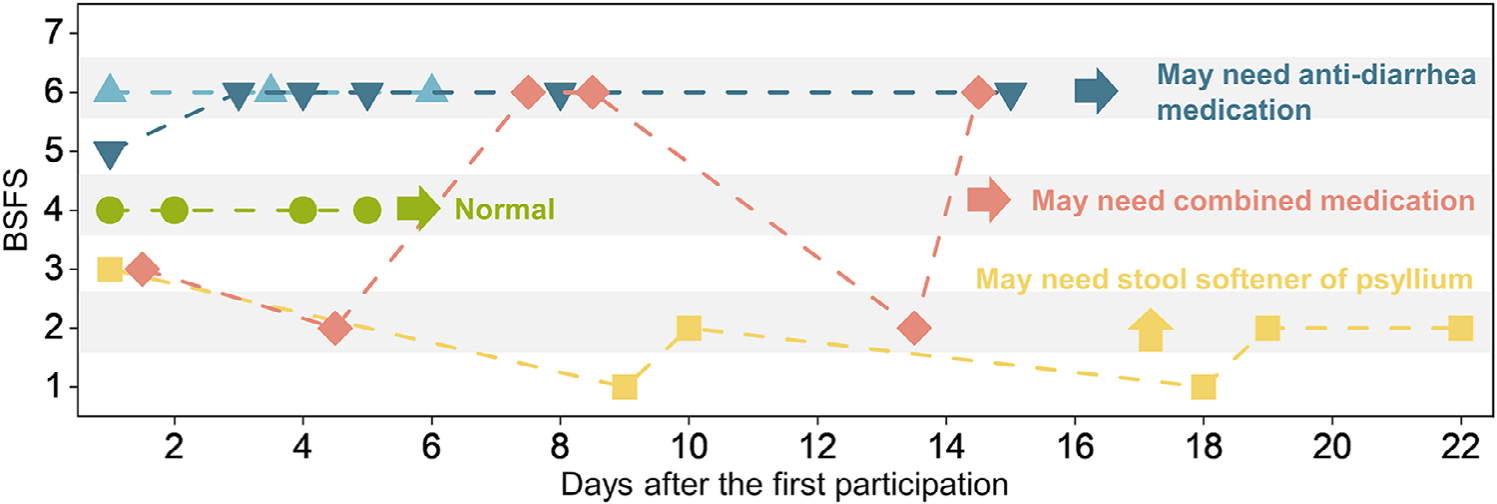
Longitudinal analysis of participant defecation patterns over a three‐week period using the smart toilet system. Each participant's defecation events are characterized by BSFS scores, with varied patterns indicating different gastrointestinal health statuses. Notably, the yellow square denotes a participant with consistently lower BSFS scores suggestive of constipation, while a couple indicated by light and dark blue triangles exhibit patterns consistent with diarrhea. The green dot represents an individual with stable and ideal stool consistency, potentially reflecting optimal gut health. In contrast, the red rhombus demonstrates fluctuating patterns, which could be indicative of an irregular bowel condition such as IBS, necessitating further medical evaluation. These visualizations aid in the potential identification of gastrointestinal health needs, informing personalized treatment recommendations.

In this study, we continuously and methodically monitored the excretory patterns of five individuals, assessing their stool consistency using BSFS. Clinicians typically recommend maintaining a stool diary for 2 to 4 weeks to diagnose conditions such as IBS. This period is sufficient to identify patterns in bowel habits, symptoms, and triggers such as diet, stress, or lifestyle factors. Shorter durations often fail to reveal episodic changes, while excessively long periods may compromise participant compliance. Based on this, we monitored the participants up to 22 days, which aligns with clinical standards and balances feasibility with data richness. Our finding, as shown in **Figure** **3**, revealed an intriguing diversity of stool patterns among participants. One participant, represented in yellow (square), consistently produced lower scores in BSFS, a pattern that may indicate constipation over a 3‐week period. Based on these observations, clinicians (or even the smart toilet system itself) could potentially offer advice such as recommending the use of over‐the‐counter stool softeners or the addition of dietary fiber like psyllium to improve stool consistency. Interestingly, two participants represented in light blue and dark blue (triangle, inverted triangle), who were identified as a couple, displayed strikingly similar patterns of continual diarrhea (BSFS 6, except for one episode). In this instance, dietary alterations or anti‐diarrhea medication might be suggested to help regulate their bowel movements. The participants symbolized in green (dot) exhibited an ideal and consistent stool pattern throughout the study period, indicating potential healthy bowel function. Meanwhile, the participant represented in the red (rhombus) demonstrated a fluctuating stool pattern over time, which could hint at medical conditions like IBS, warranting the consideration of combined medication therapy.^[^ ^28^ ^]^ It is imperative to consider that some bowel diseases, such as IBS or chronic constipation, heavily rely on symptomatology for diagnosis.^[^ ^29^ ^]^ Thus, the data from our smart healthcare toilet system could potentially offer diagnostic insights into conditions like IBS or chronic constipation by systematically tracking and analyzing bowel patterns indicative of these disorders.

It is crucial to note, however, that these observations, while informative, are purely speculative and do not constitute a medical diagnosis. For a comprehensive and accurate medical evaluation or clinical advice, a thorough medical examination by a healthcare professional is mandated. Nonetheless, these findings serve as a potential catalyst for further exploration and consultation, highlighting the potential of our method in providing valuable gut health insights. Moreover, they prove extremely useful in offering clinicians easily obtainable and objective data that can significantly aid in diagnosis and clinical management. Additionally, this systematic approach to monitoring bowel patterns could serve as an excellent preliminary screening tool for detecting gut‐related anomalies, paving the way for early intervention and management.

### 3.6 Potential Biometric Identification of Individuals

In this research, we have ventured beyond previously introduced biometric identification methods, such as fingerprint scanning and the controversial analprint recognition.^[^ ^7^ ^]^ As automatic flushing systems become more prevalent, fingerprint scanning may become less relevant, and analprint recognition has ignited significant public discourse around privacy and ethical implications. Instead, we have shifted our focus to more practical and minimally intrusive markers, such as defecation time (DT), stool drop duration (SDD), first stool drooping (FSD), activate defecation duration (ADD), total defecation duration (TDD), eu‐tenesmus duration (ETD), stool thickness (ST), Bristol stool form scale (BSFS), stool count (SC), cleansing type (CT), urination (U). A total of 11 parameters, as outlined in **Table** **1**, have been developed and classified into three categories: defecation time‐dependent parameters, stool shape‐dependent parameters, and behavior‐dependent parameters. These parameters do not include any personal data, such as gender or age, but solely pertain to defecation habits and conditions. Interestingly, our observation revealed an almost equal division between individuals who preferred to stand and those who opted to remain seated during cleaning.

**Table 1:** Information of the parameters utilized in *t*‐distributed Stochastic Neighbor Embedding (*t‐*SNE) clustering, incorporating measurements obtained from the smart toilet system and participants’ personal characteristics.

Classifications	Abbreviations	Parameters	Explanation	Data Type
Defecation time‐dependent parameters	DT	Defecation time	The precise time of the day when defecation occurs.	Int (h)
	SDD	Stool drop duration	The time elapsed from when the stool begins to descend until it contacts the water.	Float (s)
	FSD	First stool dropping	The timestamp marking the initial release of stool following seat detection.	Float (s)
	ADD	Active defecation duration	The interval from the first to the last stool drop, recorded in seconds.	Float (s)
	TDD	Total defecation duration	The total duration of time spent seated on the toilet.	Float (s)
	ETD	Eu‐tenesmus duration	The time recorded between the final stool drop and the commencement of cleansing activity	Float (s)
Stool shape‐dependent parameters	ST	Stool thickness	The width of the stool captured in the image.	Float (mm)
	BSFS	Bristol stool form scale	A scale that categorizes stool types into seven classifications, from 1 (most solid) to 7 (most liquid).	Int
	SC	Stool count	The total number of stool pieces counted per event	Int
Behavior‐dependent parameters	CT	Cleansing type	Records the posture adopted during cleansing, categorized as sitting [0] or standing [1].	Binary
	U	Urination	Documents the sequence of urination in relation to stool passage, classified as before [1] or after [0] the first stool.	Binary

To interpret these diverse parameters (as listed in Table 1), we employed a machine learning technique known as *t*‐distributed Stochastic Neighbor Embedding (*t*‐SNE). *t*‐SNE is a sophisticated computational method that facilitates the visualization of high‐dimensional data by transforming it into a more manageable two‐ or three‐dimensional space.^[^ ^30^ ^]^ This technique maintains the significant structures within the data, particularly the relationships between neighboring points, thus simplifying the dataset into a format that is more readily analyzed and interpreted. The silhouette score is a widely used metric for assessing clustering quality, providing insights into the degree of cohesion within clusters and separation between clusters. In this study, the silhouette score was utilized to evaluate how well the *t*‐SNE‐reduced data aligned with predefined participant labels, representing 11 participants labeled from 1 to 11. For a given data point *i*, the silhouette score is computed using the intracluster distance, *a*(*i*), and the inter‐cluster distance, *b*(*i*). The intra‐cluster distance *a*(*i*) is the average distance between *i* and all other points within the same cluster:

(9) $$a\left(i\right)=\frac{1}{\left|C\right|-1}\sum_{j\in C,j\neq i}d\left(i,j\right)$$

where |*C*| is the number of points with the same cluster label, and *d*(*i*,  *j*) is the distance between points *i* and *j*. The inter‐cluster distance *b*(*i*) is defined as the minimum average distance between *i* and points in other clusters:

(10) $$b\left(i\right)=\min_{C^{\prime }}\frac{1}{\left|C^{\prime }\right|}\sum_{j\in C^{\prime }}d\left(i,j\right)$$

where *C'* represents a cluster different from *i*. Using these distances, the silhouette score for a point *i* is calculated as:

(11) $$s\left(i\right)=\frac{b\left(i\right)-a\left(i\right)}{\max\left(a\left(i\right),b\left(i\right)\right)}$$

The silhouette score *s*(*i*) indicates how well the point *i* fits within its assigned cluster: *s*(*i*) ≈ 1: Point *i* is well‐aligned with its cluster. *s*(*i*) ≈ 0: Point *i* lies near the boundary between clusters. *s*(*i*) ≈ −1: Point *i* is likely assigned to the wrong cluster. The overall silhouette score is calculated as the mean silhouette score across all *n* data points:

(12) $$S=\frac{1}{n}\sum_{i=1}^{n}s\left(i\right)$$

where *n* is the total number of data points. Higher values of *S* indicate better‐defined clusters with high intra‐cluster cohesion and low inter‐cluster overlap. In this study, the silhouette score was used to evaluate the clustering results of *t*‐SNE‐reduced data. Instead of generating cluster labels using K‐means clustering, predefined participant labels ranging from 1 to 11, representing the 11 participants in the dataset, were utilized. The silhouette score was computed using the scikit‐learn library in Python with Euclidean distance as the distance metric. To preprocess the data, Min‐Max normalization was applied instead of StandardScaler, as some parameters used for *t*‐SNE, such as gender and cleansing type, are binary values. Min‐Max normalization ensured that all features were scaled within the same range without distorting the representation of binary variables. The Min‐Max normalization for a given feature *x* is defined as:

(13) $$x_{norm}=\frac{x-x_{\min}}{x_{\max}-x_{\min}}$$

where *x*_min_ and *x*_max_ are the minimum and maximum values of the feature *x*, respectively. For a given combination of features, the silhouette score was used to quantify how well the data was partitioned according to these predefined labels, enabling the selection of feature combinations that resulted in optimal clustering.

**Figure 4:**
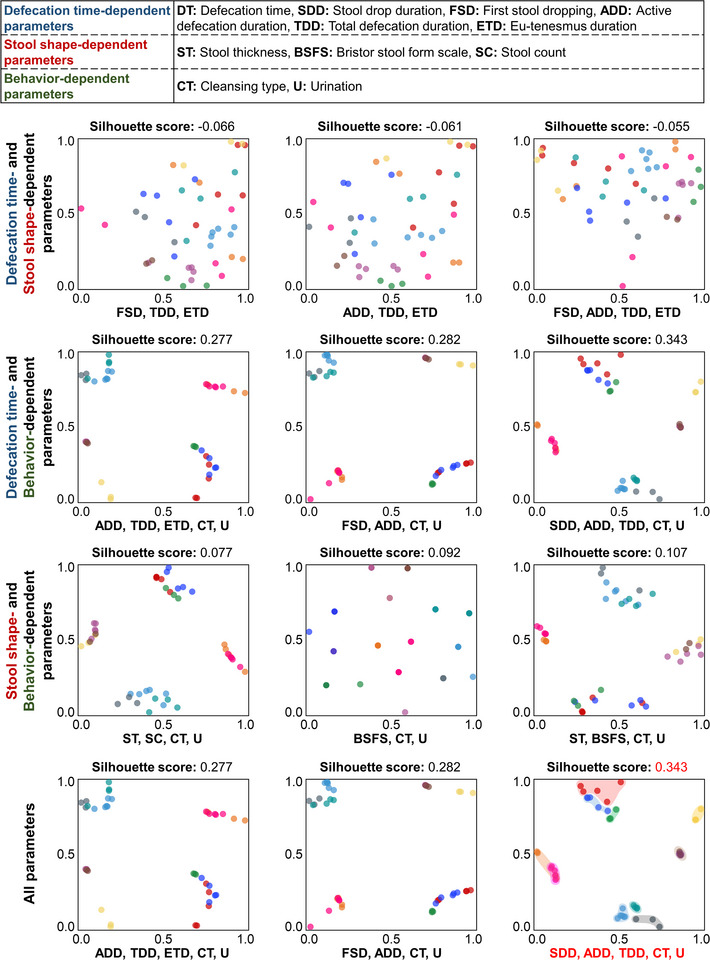
*t*‐SNE Clustering of Participants’ Defecation Characteristics (*n* = 45). Eleven defecation‐related parameters were classified into three categories (defecation time‐dependent parameters, stool shape‐dependent parameters, and behavior‐dependent parameters). For each pair of combined categories, the three highest silhouette scores for parameter combinations are shown. Additionally, the overall highest silhouette score based on all eleven parameters is also presented.

We combined two of the three parameter categories (defecation time‐dependent, stool shape‐dependent, and behavior‐dependent parameters), as well as all three together, to generate a *t*‐SNE graph that visually differentiates participants into clusters based on their unique defecation patterns. **Figure** **4** shows the three highest silhouette scores among the different category combinations.

For defecation time‐ and stool shape‐dependent parameters, the three parameter combinations with the highest silhouette scores are FSD, TDD, ETD (‐0.066); ADD, TDD, ETD (‐0.061); FSD, ADD, TDD, ETD (‐0.055). For defecation time‐ and behavior‐dependent parameters, the three parameter combinations with the highest silhouette scores are ADD, TDD, ETD, CT, U (0.277); FSD, ADD, CT, U (0.282); SDD, ADD, TDD, CT, U (0.343). For stool shape‐ and behavior‐dependent parameters, the three parameter combinations with the highest silhouette scores are ST, SC, CT, U (0.077); BSFS, CT, U (0.092); ST, BSFS, CT, U (0.107). For the combination of all parameters, the highest silhouette scores are the same as those for the defecation time‐ and behavior‐dependent parameters. A total of 1981 parameter combinations and their corresponding silhouette scores are provided in the Additional File of the *t*‐SNE analysis. We found that defecation time‐ and behavior‐dependent parameters yielded the highest silhouette scores, meaning that the clusters were more distinct from each other and well‐formed, with data points within each cluster tightly grouped together. This indicates that participants could be classified more easily based on these parameters. In contrast, combinations including stool shape‐dependent parameters tended to have lower silhouette scores. This was estimated to be because defecation time‐ and behavior‐dependent parameters are strongly linked to continuous habitual patterns, which are unique to an individual over time. In contrast, stool shape‐dependent parameters are more influenced by temporary conditions and may vary from day to day, making them less effective as biomarkers.

This approach represents a viable method for individual identification, harnessing the practicality of daily routine and the capabilities of advanced machine learning techniques. However, with only a limited number of participants in this study, we acknowledge the high risk of overfitting the model. Further studies with a larger cohort are needed to validate our findings.

## 4 Discussion

This research presents a comprehensive analysis of human defecation patterns using the smart healthcare toilet system. The richness of data harvested from participants has granted us the capacity to investigate defecation dynamics, introduce the novel concept of “eu‐tenesmus”, longitudinally track these events, and even pioneer a new approach to biometric identification. In contrast to the traditional reliance on patient‐reported outcomes—a method fraught with subjectivity—our smart toilet system facilitates the collection of objective data, offering quantifiable measures such as total defecation duration, first stool dropping time, cleansing type. These measurable factors present a window into an individual's gut health status and digestive efficiency with unprecedented clarity. The implications of this shift are profound: where once physicians had to navigate the uncertainties of self‐reported symptoms, they now have at their disposal a trove of precise data captured in real time, painting a more accurate and detailed picture of gastrointestinal well‐being. The system's innovative capability to compile and categorize user data into identifiable clusters signifies a major leap forward in personalized health monitoring, ultimately contributing to more targeted and effective treatments.

### 4.1 Clinical Implications

Our study has revealed that the time it takes for stool matter to be expelled follows a log‐normal distribution. This finding suggests that various factors, such as the viscosity of stool and its interaction with the rectal lining, contribute to the variability in expulsion times. Identifying these factors is crucial, as they could be influenced by diet, lifestyle, or underlying health conditions. This insight opens new avenues for research into how these factors can be manipulated to improve gastrointestinal health. Moreover, our data indicates that elongated falling times are usually associated with lower scores on BSFS, which could signal constipation or other digestive health issues.

Furthermore, we were able to objectively measure eu‐tenesmus in healthy individuals. eu‐tenesmus, or the sensation of incomplete bowel evacuation, has traditionally been assessed through subjective patient reports, which can be unreliable. By establishing objective baseline data, we can now more accurately diagnose and assess tenesmus, paving the way for precision medicine approaches in treating gastrointestinal disorders such as IBD and IBS. These conditions often exacerbate tenesmus, severely impacting patients’ quality of life.

For the subsequent stage of our research, we plan to investigate the impact of contemporary behaviors, specifically the usage of digital devices during defecation, on the evaluation of eu‐tenesmus. Alongside this, we will examine the post‐sitting duration after initial toilet paper use to capture the full scope of defecatory behavior, including secondary rounds of stool passage. By integrating smart toilet technology with other digital monitoring tools, we aim to meticulously track these behaviors and various contributory factors. This holistic approach will allow us to assess a comprehensive array of elements that influence gastrointestinal health. Moreover, we will investigate how interventions, such as medications or probiotics, designed to optimize the BSFS scores, affect the sensation of eu‐tenesmus. Our innovative exploration is expected to yield critical insights that may inform the development of enhanced diagnostic tools and treatment protocols. This research not only seeks to deepen our understanding of gastrointestinal health but also aims to guide personalized treatment plans that consider the subtleties of individual lifestyle factors, including post‐defecation behavior patterns and their modification following therapeutic interventions. Our study highlights the importance of understanding the physiological and behavioral factors influencing stool expulsion and tenesmus. By leveraging objective data and new technologies, we aim to improve diagnostic accuracy and treatment outcomes for individuals suffering from gastrointestinal disorders, ultimately enhancing patient care and quality of life.

The ability to monitor excretory habits longitudinally and passively has allowed us to observe a diversity of stool patterns among study participants, with potential health implications. This continuous surveillance may help detect changes and anomalies over time, leading to precision health^[^ ^5^ ^]^ including the development of personalized interventions and dietary recommendations. In the future, interconnected smart toilet systems could not only enable longitudinal and passive tracking of defecation patterns in a personalized manner but also significantly enhance public health by providing large‐scale, anonymized data insights into population‐wide trends in gastrointestinal health.^[^ ^31^ ^]^ Finally, the use of *t*‐SNE in analyzing the parameters of defecation patterns presents a viable approach to biometric identification. This serves as an alternative to traditional methods such as fingerprint scanning or analprint recognition, with potentially fewer privacy and ethical concerns.

The digitization of gut‐related events in this study represents an initial step toward enhancing our understanding of the gut‐brain axis.^[^ ^32^ ^]^ It provides new insights into the complex interactions between gastrointestinal functions and neural responses, interactions that have been challenging to quantify prior to the integration of advanced AI technologies. With this exploratory research, we begin to navigate the intricate research landscape of the gut‐brain axis, potentially contributing to a developing field in neurogastroenterology where predictive analytics might one day support the management of gut‐brain interactions.^[^ ^33^ ^]^ Such advancements could pave the way for innovative diagnostic and treatment modalities, although further investigation and validation are necessary to realize these possibilities fully.

### 4.2 Limitations

One of the significant limitations of our study was the small number of participants (*n* = 11). Additionally, the participants were aware that they were being monitored, which could possibly affect their normal toileting behaviors. This limited sample size restricts the diversity of defecation patterns and habits we could observe, and as a result, the conclusions drawn from these observations must be considered speculative rather than definitive. In addition, all participants were United States residents; thus, our findings may not be representative of the global population's defecation patterns and gut health. The findings should be interpreted cautiously, and further research with a larger, more geographically diverse cohort is necessary to validate and expand upon these results. The complexity of the algorithms used can also make their results difficult to interpret. Moreover, these algorithms require a substantial amount of data for optimal performance, which the limited sample size may not provide, potentially hindering the full potential of these techniques. Another limitation is the possibility of false‐positive or false‐negative readings due to factors such as fluctuations in light, the position of the participant, or disturbances within the toilet bowl. While the system demonstrated robust performance during the study, refinements to improve accuracy and reliability in diverse real‐world settings would be beneficial.

Another significant consideration is the potential concerns regarding user privacy and acceptance, particularly due to the presence of an optical sensor in a restroom setting. While the system is designed to capture only stool images and avoid any identifiable body parts, user apprehension about privacy remains a challenge. To mitigate this concern, we are actively investigating alternative imaging methods, such as lensless imaging, which relies on computational reconstruction from encoded optical patterns rather than forming a direct image.^[^ ^34^ ^]^ This approach not only reduces privacy risks by rendering raw image captures unintelligible without processing but also allows for a more compact and discreet hardware design. Additionally, lensless imaging maintains the capability to extract key stool characteristics, such as looseness, texture, color, volume, and shape, ensuring that clinically relevant information is preserved without compromising user comfort.

The longitudinal monitoring of excretory habits, while advantageous for observing trends and anomalies over time, raises questions about user compliance and data privacy. Ensuring user comfort and the protection of data while gathering sensitive information is paramount for the widespread adoption of this technology. Lastly, the prototype's reliance on external power and internet connectivity for data processing and storage may pose practical challenges in certain settings or regions, which could limit its applicability. Future iterations should consider integrating offline processing capabilities, such as edge computing,^[^ ^35^ ^]^ or alternative power solutions, like solar energy, to enhance the system's accessibility and utility.

## 5 Conclusion 

In conclusion, our analysis of 45 defecation events from 11 participants using a smart toilet system has provided quantitative insights into stool dropping duration, stool thickness, and an objective measure of “eu‐tenesmus.” Stool dropping duration followed a log‐normal distribution with a mean of 3.2 s (SD 3.8 s), and longer durations (>5 s) correlated with lower BSFS scores (*p* = 0.008 for BSFS1/2/3 vs BSFS5/6/7). Stool thickness averaged 26.6 mm (SD 10.9 mm), with significantly lower thickness observed at higher BSFS classifications (*p* = 5×10^−6^). We introduced the concept of eu‐tenesmus, defined as the time from the last stool drop to the commencement of cleansing. This measure had a mean of 74.8 s (SD 74.9 s) and showed a statistically significant difference between female and male participants (*p* = 0.014), though no significant correlation with BSFS was detected. Eu‐tenesmus has the potential to serve as an important digital biomarker for assessing gut health, independent of stool condition.

Preliminary biometric analyses incorporating 11 defecation‐related parameters indicated that time‐ and behavior‐dependent parameters played a more significant role in participant classification than stool shape‐dependent parameters. The use of the smart healthcare toilet system in monitoring and analyzing human defecation habits has opened new avenues for personalized gut health monitoring, diagnostics, and timely medical intervention. Despite societal taboos surrounding this subject, it is important to recognize the potential of these advancements, as they can aid in disease diagnosis, improve health outcomes, and enhance overall quality of life. Future research should continue to explore these possibilities, ensuring that technological advancements are effectively leveraged to maximize their benefits for human gut health.

## Conflict of Interest

S.‐m.P. and D.D.W. are co‐founders of a stealth mode start‐up (Kanaria Health) specializing in the implemented of a similar technology developed in the manuscript.

## Author Contributions

Z.S., T.H.K., and J.L. contributed equally to this work. Z.S., T.H.K., J.L., and S.‐M.P. performed data analysis and wrote the initial draft of the manuscript. B.J.L. recruited participants, constructed the hardware and software, and reviewed the manuscript. D.D.W., H.S.C., J.C.L., W.G.P., I.S., S.R., M.J.R., J.K.Z., and S.H.W. conducted the clinical review of the manuscript. B.H.J. and S.K. provided critical discussion on data interpretation and facilitated the original idea. D.L.H. provided animal data and contributed to the discussion of the data. S.‐M.P. provided administrative and material support, supervision, critical review, and editing of the manuscript. All authors read and approved the final manuscript.

## Additional Information

All study participants provided informed consent, and the study protocol was approved by the relevant ethical committee (Stanford IRB Protocol #45 621). All data was anonymized and handled according to strict privacy and data protection guideline.

## Supporting information



Supporting Information

Supporting Information

## Data Availability

The data that support the findings of this study are available on request from the corresponding author. The data are not publicly available due to privacy or ethical restrictions.
