# Supplementary material for: AI‐Driven Defecation Analysis by Smart Healthcare Toilet: Exploring Biometric Patterns and Eu‐Tenesmus
Source: Adv Sci (Weinh). 2025 May 11;12(30):2503247. doi: 10.1002/advs.202503247 (PMC12376671; doi:10.1002/advs.202503247)
Supplement: Supplementary file 1 — Supporting Information [file ADVS-12-2503247-s002.docx]

**AI-DRIVEN DEFECATION ANALYSIS BY SMART HEALTHCARE TOILET: EXPLORING BIOMETRIC PATTERNS AND EU-TENESMUS**

Zhiquan Song^1^†, TaeHyung Kwon^2^†, Jeung Lee^2^†, Daeyoun D. Won^3,4^, Brian J. Lee^5^, Hyuk Soon Choi^6^, Joseph C. Liao^7^, Walter G. Park^8^, Irene Sonu^8^, Stephan Rogalla^8^, Michael J. Rosen^9^, David L. Hu^10^, Jonathan Kuang Ziyang^11^, Sunny Hei Wong^11,12^, Bong Hyun Jun^13,^*, Soh Kim^2,^*, Seung-min Park^1,12^*

^1^School of Chemistry, Chemical Engineering and Biotechnology, Nanyang Technological University, Singapore, Singapore.

^2^Department of Civil and Environmental Engineering, Stanford University, Stanford, CA, USA.

^3^Seokjeong Wellpark Hospital, Jeollabuk-do, Republic of Korea.

^4^Kanaria Health, Seoul, Republic of Korea.

^5^School of Mechanical Engineering, Sungkyunkwan University, Suwon, Republic of Korea.

^6^Division of Gastroenterology and Hepatology, Department of Internal Medicine, Korea University College of Medicine, Seoul, Republic of Korea.

^7^Department of Urology, Stanford University School of Medicine, Stanford, CA, USA.

^8^Division of Gastroenterology & Hepatology, Department of Medicine, Stanford University School of Medicine, Stanford, CA, USA.

^9^Division of Pediatric Gastroenterology, Hepatology and Nutrition, Department of Pediatrics, Stanford University School of Medicine, Stanford, CA, USA.

^10^George W. Woodruff School of Mechanical Engineering, Georgia Institute of Technology, GA, USA.

^11^Department of Gastroenterology & Hepatology, Tan Tock Seng Hospital, Singapore.

^12^Lee Kong Chian School of Medicine, Nanyang Technological University, Singapore, Singapore.

^13^Department of Bioscience and Biotechnology, Konkuk University, Seoul, Republic of Korea.

†These authors are equally contributed to the work.

**Corresponding Author Contact Information:**

*Bong-Hyun Jun

Email: [bjun@konkuk.ac.kr](mailto:bjun@konkuk.ac.kr)

*Soh Kim

Email: [sohkim@stanford.edu](mailto:sohkim@stanford.edu)

*Seung-min Park

Postal address: School of Chemistry, Chemical Engineering and Biotechnology, Nanyang Technological University, 62 Nanyang Drive, N1.3-B2-08, Singapore 637459.

Email: [park.seungmin@ntu.edu.sg](mailto:park.seungmin@ntu.edu.sg)

**Table S1**. **Analysis of participant data across various parameters.**

Table presents a comprehensive analysis of participant data across multiple trials. The table includes metrics such as defecation time, stool drop duration, and stool thickness, among others, recorded for each participant. The columns represent different episodes, while the rows provide detailed measurements for each parameter. One of the variables, the Bristol Stool Form Scale (BSFS) has 4 values separated by commas. This is because, the current study employs 4 different evaluations of BSFS, 2 from manual evaluations by 2 colorectal surgeons and 2 from distinctive artificial intelligence models.

| **Participant** | **Trial**  **(n)** | **Defecation Time** | **Stool Drop Duration**  **(Sec)** | **Stool Thickness**  **(Pixel)** | **First Stool Dropping**  **(Sec)** | **Active Defecation Duration**  **(Sec)** | **Total Defecation Duration**  **(Sec)** |
| --- | --- | --- | --- | --- | --- | --- | --- |
| 1 | 0 | 9 | 0.6682 | 221.60 | 1.74 | 85.89 | 135.93 |
| 1 | 1 | 8 | 2.0226 | 239.00 | 3.70 | 79.75 | 128.61 |
| 1 | 6 | 6 | 1.5264 | 246.00 | 4.60 | 89.62 | 132.42 |
| 1 | 7 | 6 | 2.0415 | 182.33 | 8.22 | 74.01 | 148.00 |
| 1 | 9 | 6 | 1.1438 | 70.29 | 12.19 | 92.08 | 134.63 |
| 1 | 10 | 8 | 0.5073 | 151.67 | 1.00 | 159.75 | 163.36 |
| 2 | 1 | 18 | 6.7895 | 207.67 | 4.54 | 328.00 | 161.00 |
| 2 | 3 | 22 | 3.5434 | 296.33 | 9.72 | 326.28 | 318.00 |
| 2 | 4 | 3 | 15.1285 | 304.80 | 29.58 | 114.13 | 143.71 |
| 2 | 5 | 22 | 7.0501 | 304.80 | 40.93 | 131.70 | 172.63 |
| 2 | 6 | 17 | 4.8807 | 296.00 | 16.91 | 372.02 | 388.93 |
| 3 | 1 | 8 | 1.1373 | 136.67 | 2.57 | 42.23 | 55.00 |
| 3 | 2 | 7 | 4.1904 | 194.33 | 15.24 | 32.72 | 66.80 |
| 3 | 3 | 7 | 10.2154 | 165.00 | 20.50 | 274.93 | 253.98 |
| 3 | 4 | 8 | 6.4463 | 165.94 | 33.03 | 307.51 | 376.49 |
| 4 | 1 | 9 | 1.1226 | 174.00 | 4.63 | 22.38 | 41.88 |
| 4 | 2 | 9 | 1.3543 | 115.80 | 15.59 | 116.00 | 68.50 |
| 4 | 3 | 9 | 5.2309 | 206.40 | 97.30 | 141.16 | 178.43 |
| 4 | 5 | 10 | 1.9788 | 157.38 | 203.98 | 92.40 | 296.38 |
| 4 | 6 | 10 | 3.2571 | 142.50 | 18.63 | 123.46 | 143.30 |
| 5 | 1 | 9 | 1.1195 | 103.33 | 8.02 | 379.66 | 348.77 |
| 5 | 2 | 9 | 1.1723 | 202.33 | 2.34 | 362.02 | 374.30 |
| 5 | 3 | 10 | 1.8303 | 202.00 | 9.59 | 514.41 | 524.00 |
| 6 | 1 | 10 | 1.6113 | 209.00 | 15.09 | 43.57 | 56.31 |
| 6 | 2 | 16 | 3.6239 | 213.29 | 47.75 | 44.57 | 92.32 |
| 6 | 3 | 10 | 6.7428 | 334.00 | 19.58 | 39.70 | 76.58 |
| 6 | 4 | 13 | 7.9017 | 313.33 | 18.70 | 57.41 | 76.11 |
| 6 | 5 | 10 | 2.1826 | 370.33 | 15.13 | 57.62 | 72.75 |
| 7 | 1 | 13 | 8.2072 | 428.67 | 53.52 | 134.54 | 198.55 |
| 7 | 2 | 13 | 3.0257 | 351.50 | 58.62 | 59.26 | 224.62 |
| 7 | 3 | 13 | 11.7988 | 321.00 | 22.76 | 87.56 | 148.53 |
| 8 | 1 | 10 | 2.6768 | 333.50 | 27.37 | 167.18 | 206.63 |
| 8 | 2 | 9 | 2.3356 | 214.75 | 58.37 | 155.81 | 214.18 |
| 8 | 3 | 9 | 3.2358 | 130.80 | 14.80 | 134.93 | 149.73 |
| 8 | 4 | 9 | 1.4895 | 130.80 | 10.80 | 209.99 | 230.79 |
| 9 | 1 | 11 | 3.1642 | 305.67 | 32.67 | 149.73 | 186.16 |
| 9 | 2 | 9 | 4.5832 | 252.33 | 29.80 | 238.33 | 268.13 |
| 9 | 3 | 15 | 4.8933 | 314.00 | 25.59 | 84.75 | 110.34 |
| 9 | 4 | 16 | 3.2492 | 473.00 | 13.51 | 257.46 | 270.97 |
| 9 | 5 | 16 | 3.6220 | 333.50 | 50.38 | 233.28 | 283.66 |
| 10 | 2 | 9 | 1.9706 | 310.00 | 8.79 | 21.22 | 30.01 |
| 10 | 3 | 9 | 1.8881 | 172.50 | 22.10 | 8.67 | 30.77 |
| 10 | 4 | 9 | 7.0756 | 211.25 | 33.25 | 20.47 | 53.72 |
| 11 | 1 | 9 | 2.5254 | 220.67 | 43.11 | 97.11 | 101.02 |
| 11 | 2 | 8 | 6.6834 | 108.33 | 46.07 | 53.79 | 99.86 |

| **Participant** | **Trial**  **(n)** | **eu-tenesmus Duration**  **(Sec)** | **BSFS**  **(1-7)** | **Gender** | **Cleansing Type** | **Urination** | **Age** | **Stool Count**  **(n)** |
| --- | --- | --- | --- | --- | --- | --- | --- | --- |
| 1 | 0 | 36.37 | 4,4,4,4 | 1 | 1 | 0 | 40 | 8 |
| 1 | 1 | 68.30 | 4,4,3,4 | 1 | 1 | 0 | 40 | 3 |
| 1 | 6 | 80.45 | 3,4,6,3 | 1 | 1 | 0 | 40 | 1 |
| 1 | 7 | 65.28 | 4,3,6,5 | 1 | 1 | 0 | 40 | 3 |
| 1 | 9 | 56.41 | 4,4,4,4 | 1 | 1 | 0 | 40 | 5 |
| 1 | 10 | 49.03 | 6,6,6,6 | 1 | 1 | 0 | 40 | 3 |
| 2 | 1 | 137.42 | 3,3,3,3 | 0 | 0 | 1 | 41 | 2 |
| 2 | 3 | 301.70 | 2,2,2,2 | 0 | 0 | 1 | 41 | 2 |
| 2 | 4 | 66.66 | 2,2,2,2 | 0 | 0 | 1 | 41 | 1 |
| 2 | 5 | 123.42 | 2,2,2,2 | 0 | 0 | 1 | 41 | 1 |
| 2 | 6 | 257.34 | 2,2,2,6 | 0 | 0 | 1 | 41 | 1 |
| 3 | 1 | 67.52 | 4,4,4,4 | 1 | 0 | 0 | 30 | 5 |
| 3 | 2 | 62.68 | 4,4,4,4 | 1 | 0 | 0 | 30 | 1 |
| 3 | 3 | 110.44 | 4,4,4,4 | 1 | 0 | 0 | 30 | 3 |
| 3 | 4 | 100.33 | 4,4,4,4 | 1 | 0 | 0 | 30 | 12 |
| 4 | 1 | 28.81 | 5,5,5,5 | 1 | 0 | 0 | 35 | 5 |
| 4 | 2 | 41.74 | 6,6,6,6 | 1 | 0 | 0 | 35 | 6 |
| 4 | 3 | 19.85 | 6,6,6,6 | 1 | 0 | 0 | 35 | 10 |
| 4 | 5 | 57.49 | 5,5,6,5 | 1 | 0 | 0 | 35 | 7 |
| 4 | 6 | 107.03 | 5,5,6,5 | 1 | 0 | 0 | 35 | 6 |
| 5 | 1 | 105.87 | 3,5,6,6 | 0 | 1 | 1 | 26 | 5 |
| 5 | 2 | 293.88 | 4,4,6,6 | 0 | 1 | 1 | 26 | 3 |
| 5 | 3 | 237.37 | 3,3,4,4 | 0 | 1 | 1 | 26 | 9 |
| 6 | 1 | 5.80 | 5,5,6,6 | 1 | 1 | 1 | 28 | 6 |
| 6 | 2 | 14.55 | 3,4,3,3 | 1 | 1 | 1 | 28 | 1 |
| 6 | 3 | 17.66 | 3,3,1,6 | 1 | 1 | 1 | 28 | 2 |
| 6 | 4 | 18.91 | 3,3,3,5 | 1 | 1 | 1 | 28 | 4 |
| 6 | 5 | 12.62 | 3,3,3,4 | 1 | 1 | 1 | 28 | 4 |
| 7 | 1 | 41.30 | 3,3,2,2 | 0 | 1 | 0 | 19 | 2 |
| 7 | 2 | 34.25 | 2,2,2,2 | 0 | 1 | 0 | 19 | 2 |
| 7 | 3 | 26.14 | 4,4,5,5 | 0 | 1 | 0 | 19 | 5 |
| 8 | 1 | 95.53 | 5,5,6,6 | 0 | 1 | 0 | 29 | 8 |
| 8 | 2 | 109.88 | 3,4,5,5 | 0 | 1 | 0 | 29 | 6 |
| 8 | 3 | 31.70 | 4,4,5,5 | 0 | 1 | 0 | 29 | 7 |
| 8 | 4 | 180.80 | 5,5,6,6 | 0 | 1 | 0 | 29 | 10 |
| 9 | 1 | 45.14 | 3,3,3,3 | 1 | 0 | 1 | 22 | 4 |
| 9 | 2 | 49.20 | 2,2,2,2 | 1 | 0 | 1 | 22 | 7 |
| 9 | 3 | 18.17 | 3,4,6,6 | 1 | 0 | 1 | 22 | 5 |
| 9 | 4 | 104.79 | 3,4,6,6 | 1 | 0 | 1 | 22 | 4 |
| 9 | 5 | 46.74 | 2,2,2,5 | 1 | 0 | 1 | 22 | 3 |
| 10 | 2 | 7.45 | 2,2,2,2 | 0 | 0 | 1 | 31 | 2 |
| 10 | 3 | 3.71 | 4,4,4,4 | 0 | 0 | 1 | 31 | 2 |
| 10 | 4 | 8.55 | 3,3,2,2 | 0 | 0 | 1 | 31 | 2 |
| 11 | 1 | 12.74 | 5,5,6,5 | 1 | 1 | 1 | 29 | 3 |
| 11 | 2 | 3.59 | 5,5,6,5 | 1 | 1 | 1 | 29 | 4 |


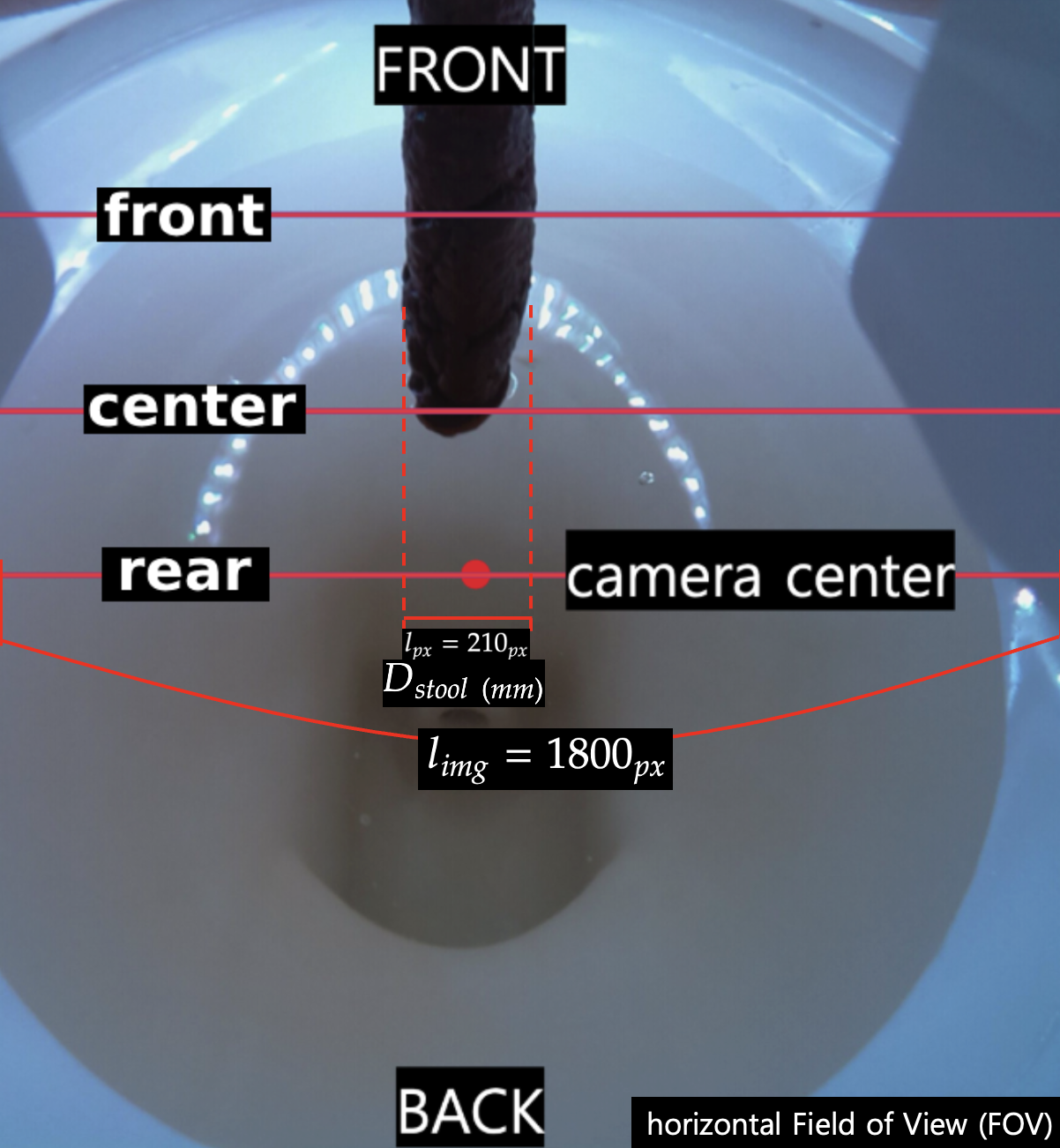


**Figure S1. Schematic illustration of stool thickness measurement within the smart toilet system.** The image shows a top-down view of the toilet bowl, with three representative stool-drop locations labeled (front, center, rear). The horizontal red lines indicate the approximate regions where the stool may drop relative to the participant’s seating position, and the central red dot marks the camera’s optical center. The total horizontal field of view (FOV) spans approximately 1800 pixels. In this example, the stool’s diameter (in pixels) is converted to millimeters by applying a transfer coefficient that accounts for the camera’s distance and angle. This setup allows for consistent measurement of stool thickness under real-world usage conditions.
